# CitGVD: a comprehensive database of citrus genomic variations

**DOI:** 10.1038/s41438-019-0234-3

**Published:** 2020-02-01

**Authors:** Qiang Li, Jingjing Qi, Xiujuan Qin, Wanfu Dou, Tiangang Lei, Anhua Hu, Ruirui Jia, Guojin Jiang, Xiuping Zou, Qin Long, Lanzhen Xu, Aihong Peng, Lixiao Yao, Shanchun Chen, Yongrui He

**Affiliations:** 1 Citrus Research Institute, Southwest University/Chinese Academy of Agricultural Sciences, 400712 Chongqing, China; 2 Key Laboratory of Plant Hormones and Development Regulation of Chongqing, School of Life Sciences, Chongqing University, 401331 Chongqing, China

**Keywords:** Genetic markers, Structural variation

## Abstract

Citrus is one of the most important commercial fruit crops worldwide. With the vast genomic data currently available for citrus fruit, genetic relationships, and molecular markers can be assessed for the development of molecular breeding and genomic selection strategies. In this study, to permit the ease of access to these data, a web-based database, the citrus genomic variation database (CitGVD, http://citgvd.cric.cn/home) was developed as the first citrus-specific comprehensive database dedicated to genome-wide variations including single nucleotide polymorphisms (SNPs) and insertions/deletions (INDELs). The current version (V1.0.0) of CitGVD is an open-access resource centered on 1,493,258,964 high-quality genomic variations and 84 phenotypes of 346 organisms curated from in-house projects and public resources. CitGVD integrates closely related information on genomic variation annotations, related gene annotations, and details regarding the organisms, incorporating a variety of built-in tools for data accession and analysis. As an example, CitGWAS can be used for genome-wide association studies (GWASs) with SNPs and phenotypic data, while CitEVOL can be used for genetic structure analysis. These features make CitGVD a comprehensive web portal and bioinformatics platform for citrus-related studies. It also provides a model for analyzing genome-wide variations for a wide range of crop varieties.

## Introduction

Citrus is grown in more than 100 countries. The worldwide production and total acreage of citrus fruits ranks first among all fruit crops. The most widely cultivated citrus species under domestication and selective breeding include sweet orange (*Citrus sinensis*), mandarin (*Citrus reticulata*), pummelo (*Citrus grandis*), grapefruit (*Citrus paradisi*), and lemon (*Citrus limon*)^1,2^. Draft genome sequences of several citrus species have been released in genomic databases citrus annotation project (CAP)^3^ and Phytozome^4,5^. With the availability of reference genomes^1,2,4,6^, bulk data from citrus resequencing projects have been compiled and can be applied for population genetics, including genome-wide association studies (GWASs)^7^, evolutionary studies^2^, and comparative genomics. These have identified key genomic variations that have led to the discovery of key quantitative trait loci (QTLs), molecular genetic markers or genes relevant to important traits and contribute to our understanding of citrus origin and evolutionary relationships.

Single nucleotide polymorphisms (SNPs) and insertions/deletions (INDELs) have been widely employed in citrus breeding^8^. Genetic variations are considered to be molecular markers and improve our understanding of the genetic basis of phenotypic variations observed in many agronomic traits via linkage and association mapping^9,10^. To date, the rapid development of next-generation sequencing (NGS) technologies has facilitated the generation of large citrus datasets^2^. However, the identification of key SNPs/INDELs from the large NGS datasets is laborious and requires extensive computational resources. Current SNP/INDEL datasets are not user friendly, and more comprehensive databases/platforms that focus on citrus genomic variations are required. Such a platform should include abundant data on high-quality genomic variations and detailed genotype/phenotype information for an abundance of citrus accessions. The system should also contain a user-friendly interface to analyze and visualize data.

GWASs have led to the discovery of a large number of genetic loci associated with different species traits, including those in peaches^11^, rice^12^, wheat^13^, sorghum^14^, and dogs^15^, based on whole-genome variations. The genotyping process before GWASs, association analysis in GWASs, and molecular markers and functional gene analysis after GWASs are indispensable for genome-wide and evolutionary analysis. To ensure the efficient use of these data, several genomic variation databases have been developed, including DoGSD for dogs^16^, SorGSD for sorghum^17^, and RiceVarMap^18^ for rice.

Herein, we present CitGVD (http://citgvd.cric.cn/home), a comprehensive database of citrus genomic variations that provides a publicly available and free data service for scientific studies. Currently, CitGVD includes large sets of data on genomic variations (SNPs and INDELs) compiled from two released reference genomes for *Citrus clementina* and *Citrus grandis*^2^, including 84 phenotypes, gene functional annotations and informative literature. CitGVD also provides in-depth analysis, including CitTRAIT for phenotypic data statistics, CitGWAS for GWASs based on built-in data, CitEVOL for genetic evolution analysis, PCR primer design and Gbrowse for variations and genes. All data including genotypic data, phenotypic data, bulk NGS data and reference data can be accessed or downloaded freely, and the built-in tools from CitGVD can be used free of charge with computing resources powered by the database creator.

CitGVD therefore represents a specialized repository of public sequences and data repositories deduced from in-house pipelines. CitGVD provides unique and powerful tools for further in-depth analysis as opposed to a simple “database”. The combination of these features makes CitGVD a comprehensive web portal and bioinformatics platform for citrus-related studies across the global research community. It also provides a model for analyzing genome-wide variations and building variation databases for a wider range of crop varieties.

## Database construction

### Implementation

The free and popular relational database management system MySQL^19^ and the J2EE framework were used to develop CitGVD (V1.0.0). Modern user interfaces were developed using JavaServer Pages (JSP), HTML5, and CSS3. The tools built-in CitGVD were compiled with Perl (V3.5.6) and operated in the Linux environment. Gbrowse^20^ (V2.54) was integrated for chromosome-based genomic variation and gene visualization, while Primer Design (V4.1.0) can retrieve primers for citrus SNPs, INDELs, and genes. BLAST (V2.2.31) is used to search the orthologs or paralogs of the input sequences in CitGVD.

### Data sources and processing

**Fig. 1:**
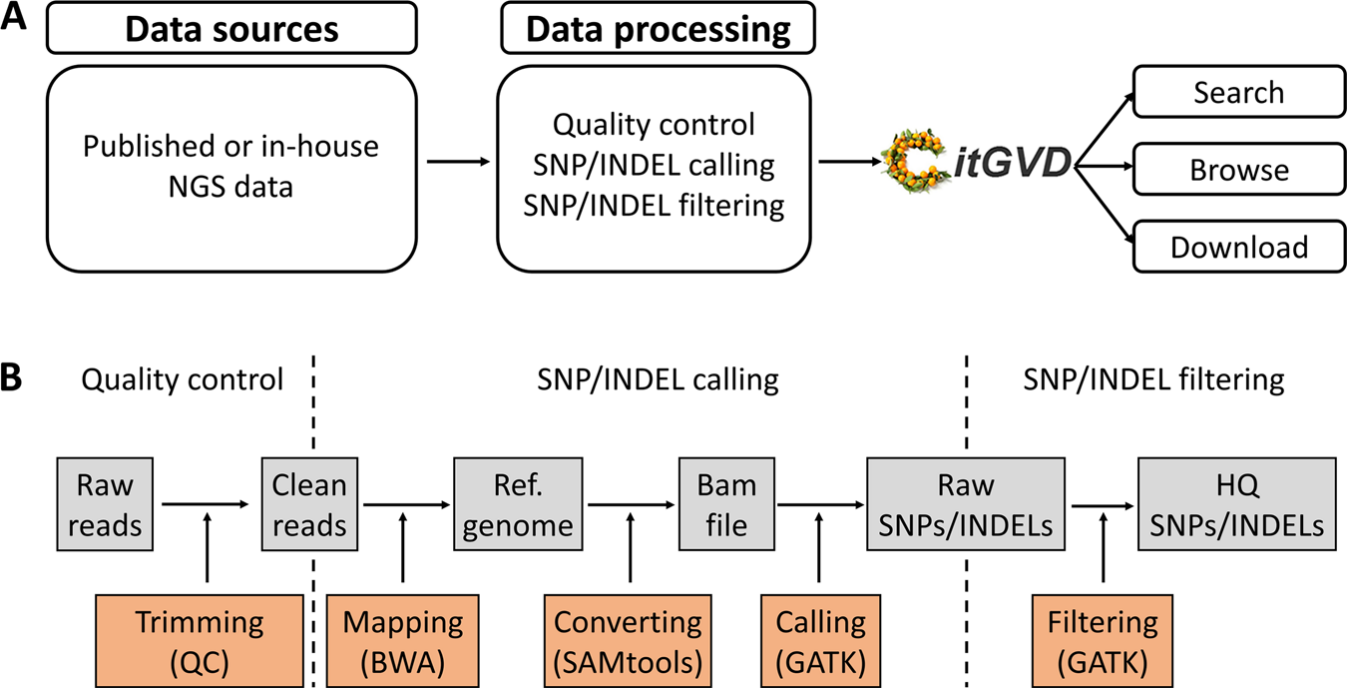
Workflow highlighting the development of CitGVD. **a** Data sources and pipelines to construct CitGVD. **b** Data processing procedures. This process included NGS data quality control, SNP calling and filtering.

The construction of CitGVD was a multistep process. The raw paired-end reads from in-house or previously published NGS data were prepared and processed with the in-house pipeline (Fig. 1a). With 1,493,258,964 nonredundant variations from 346 citrus species, a web interface was designed to search, browse, download, and analyze the built-in data, together with phenotypic and gene annotation analysis. In total, 84 phenotypes were evaluated, and genes of *Citrus clementina* (CCL)^4,5^ and *Citrus grandis* (CGR)^6^ were annotated based on kyoto encyclopedia of genes and genomes (KEGG)^21^, gene ontology (GO)^22^, eukaryotic orthologous groups (KOG)^23^, NCBI’s nonredundant protein database (Nr)^24^, and translated EMBL (TrEMBL)^25^ and included in CitGVD. For data processing, raw reads were trimmed and mapped to the reference citrus genomes using the BWA program (V0.6.2)^26^. The SAMtools package^27^ was used to convert the mapping results to the BAM format. Finally, SNPs/INDELs were identified and filtered using GATK (V2.5.0)^28,29^ (Fig. 1b).

## Usage and access

CitGVD offers four functional sections: “BROWSE”, “SEARCH”, “TOOLS”, and “DOWNLOAD”, centralized as citrus genomic variations and two sections termed “GENERAL” and “HELP” for a detailed introduction and user tutorials. These built-in functional modules can not only work independently but also cooperate with each other.

### BROWSE

**Fig. 2:**
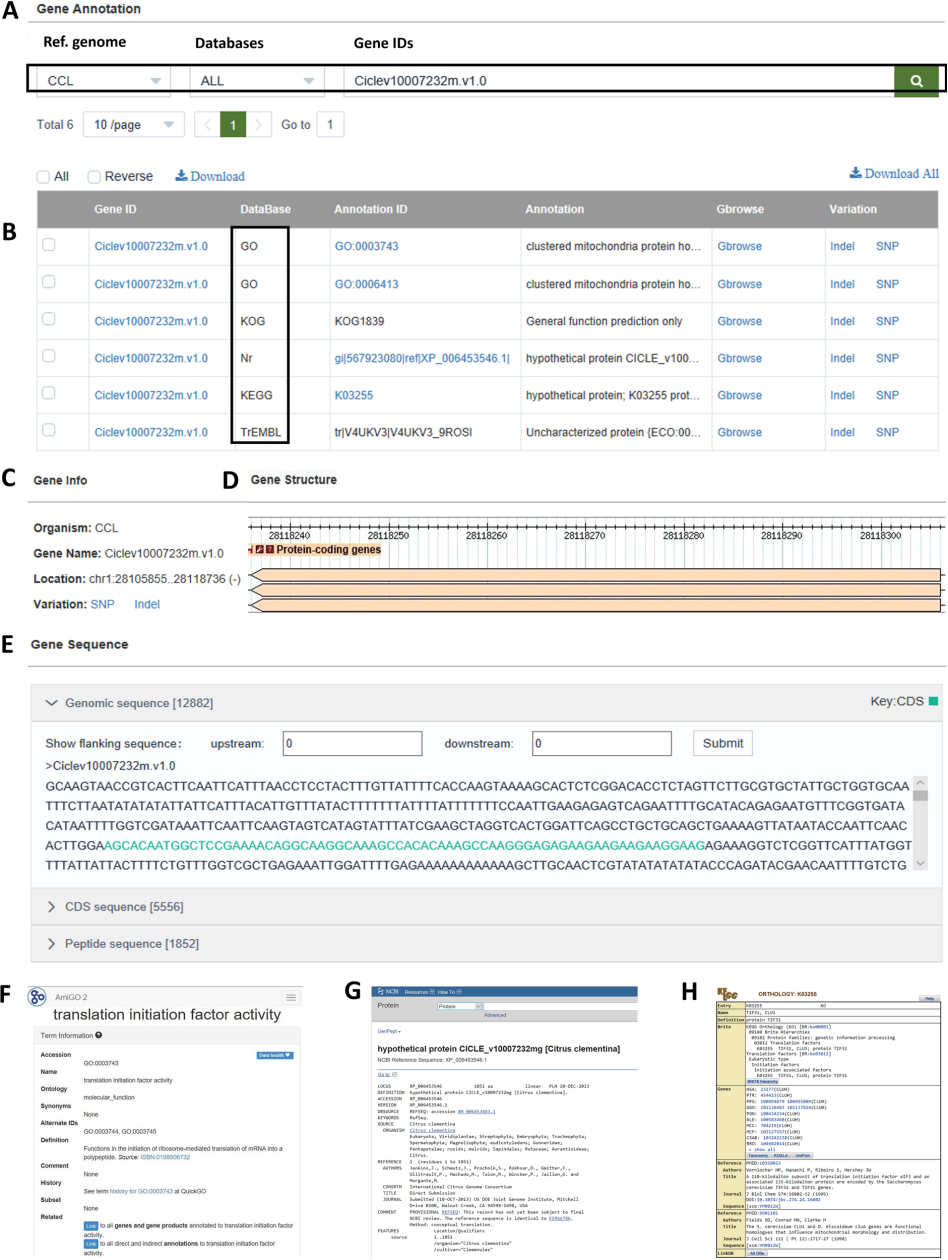
Screen dumps of the gene browse function of CitGVD. **a** Three query strategies by ref. genomes, annotation sources, and gene IDs, respectively, can be used for data filtering. **b** Annotations from five sources including KEGG, GO, Nr, KOG, and TrEMBL can be retrieved in CitGVD. Gene information (**c**), gbrowse visualizations (**d**), genomic sequences, CDS, peptide sequences (**e**) can be accessed by the cross-link on the gene ID. The annotation details of GO (**f**), Nr (**g**), and KEGG (**h**) can be accessed by the cross-links on corresponding annotation IDs (**b**).

Under the “BROWSE” pull-down menu, CitGVD provides data browsing functionalities, including SNP/INDEL browse, designed for the statistics of genomic variations according to chromosomes, types and germplasms, gene annotation browse for gene information and annotations, and germplasm browse for browsing germplasm details, including germplasm ID, name, origin, type and other information. On the gene browse page, the data can be filtered according to two reference species CCL and CGR, five annotation sources or entered gene IDs (Fig. 2a). In CitGVD, the genes of the two reference species were annotated by KEGG^21^, GO^22^, Nr^24^, KOG^23^, and TrEMBL^25^ (Fig. 2b). By clicking on the hyperlink associated with the gene ID, a new page can be opened, and gene information (Fig. 2c), gbrowse visualizations (Fig. 2d), genomic sequences, coding sequences (CDSs), and peptide sequences (Fig. 2e) can be accessed. In the current version, gene annotations from KEGG, Nr, and GO were cross-linked to the source databases (Fig. 2b). By clicking on the annotation IDs, users can navigate to the source pages for original annotation details (Fig. 2f–h).

### SEARCH

**Fig. 3:**
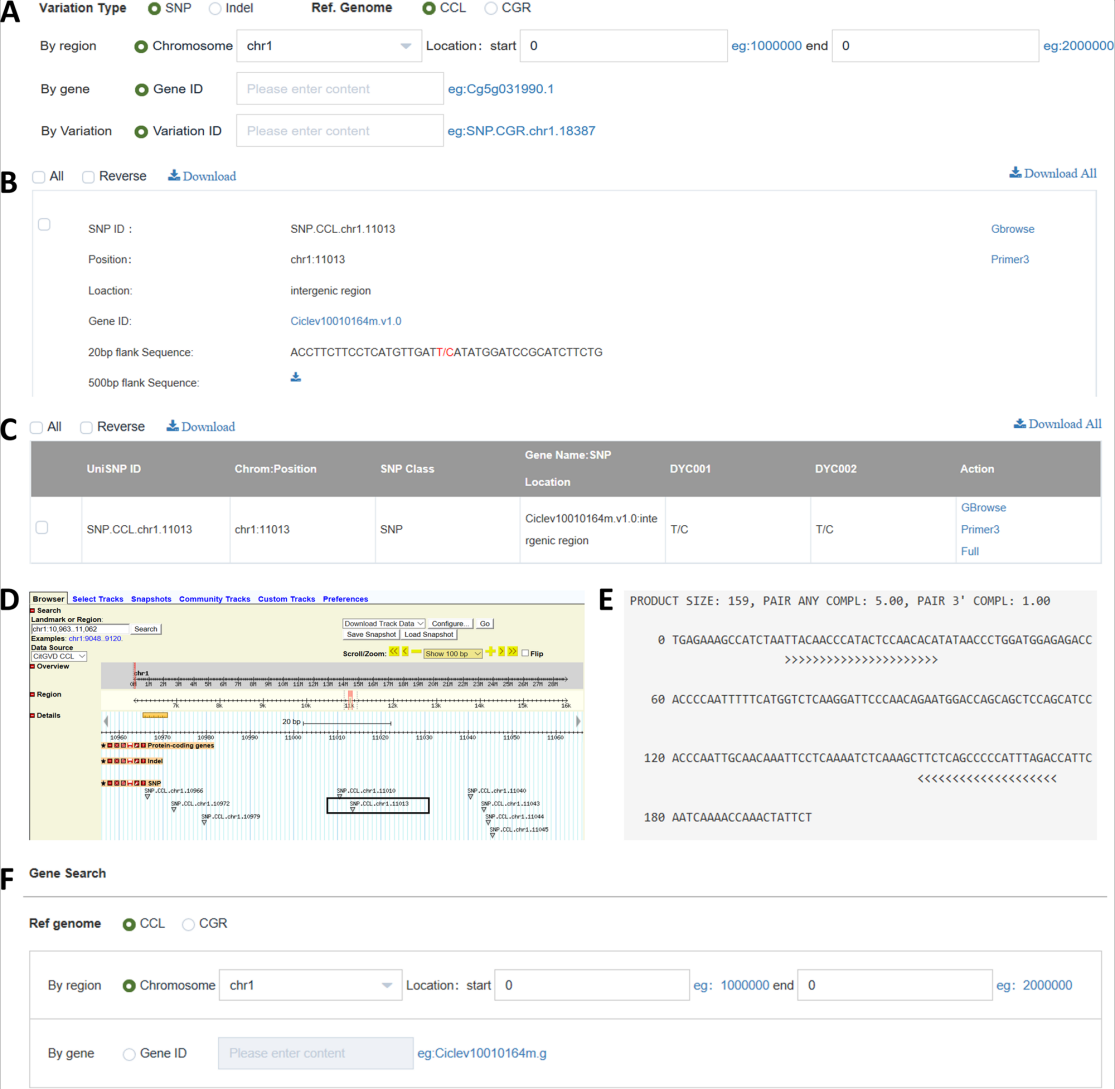
Screen dumps of the SNP/INDEL search functions of CitGVD. **a** Three query strategies for SNP/INDEL searches. **b** A typical search result of the Multicriteria Search. **c** A typical search result of the Comparative Search. **d** Gbrowse visualization of a SNP. **e** Primer Design for a SNP. **f** Gene Search tool for a SNP/INDEL-related gene.

The “SEARCH” function provides a user-friendly web interface to query SNP/INDEL information by specifying the chromosomal start and end loci, gene ID and SNP/INDEL IDs (Fig. 3a). SNP/INDEL searches for one individual (Multicriteria Search) (Fig. 3b) and comparative searches of SNP/INDELs between two or more individuals (Comparative Search) (Fig. 3c) are implemented in CitGVD. In the results of the Multicriteria Search, SNP/INDEL IDs, chromosomal positions, locations, related genes and up- or downstream flanking sequences are displayed (Fig. 3b), while in the results of the Comparative Search, SNP/INDEL IDs, chromosomal positions, locations, and related genes are directly displayed (Fig. 3c). In both search strategies, guided by a hyperlink directing Gbrowse, the SNP/INDEL sites can be chromosomally visualized (Fig. 3d). For bench researchers, primers for the SNP/INDEL sites can be used to validate the NGS data or to perform molecular marker development. The built-in Primer Design tool can be easily accessed via the hyperlink on the results page (Fig. 3e). Users can also navigate to a detailed page of SNP/INDEL-related genes with the cross-link on the gene IDs (Fig. 3b). The gene details can also be retrieved via the Gene Search tool by searching the IDs of SNP/INDEL-related genes (Fig. 3f). There is also a Phenotype Search set in CitGVD to search and retrieve phenotypic data pertaining to fruit-related traits, floral traits, leaf-related traits and other traits for species in CitGVD.

### TOOLS

**Fig. 4:**
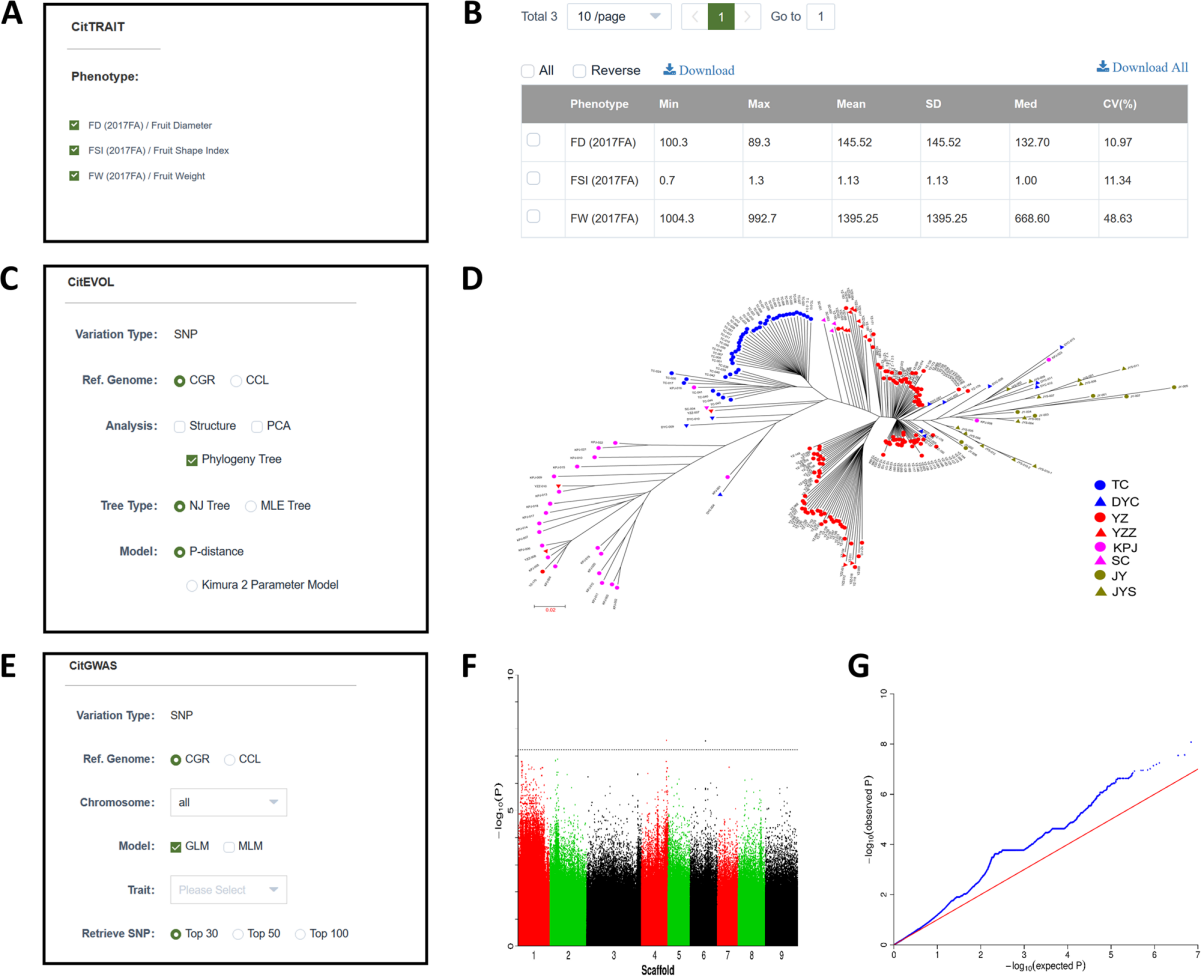
Built-in pipelines. **a** Parameters of CitTRAIT. **b** Output of CitTRAIT. Min minimum value, Max maximum value, Mean mean value, SD standard deviation, Med median, CV coefficient of variation. **c** Parameters of CitEVOL. CitEVOL processes with built-in SNP data. In the calculation, the reference genome was first determined, and then the population structure, principal component analysis (PCA) results and phylogenetic trees can be analyzed. **d** Phylogenetic tree data were assessed with CitEVOL and visualized with MEGA V7.2. **e** Parameters of CitGWAS. Mmixed linear model (MLM) and general linear model (GLM) can be used for GWASs. **f** Manhattan plot output from CitGWAS. **g** QQ plot output from CitGWAS.

To highlight CitGVD as more than just a database, deep analysis tools and pipelines were developed. “TOOLS” contains CitTRAIT, CitEVOL, and CitGWAS, three pipelines committed to phenotypic statistics, genetic relationships, and GWASs to mine trait-related molecular markers that will benefit citrus molecular breeding. To initiate CitGWAS, a run of CitTRAIT is performed to check the variation in traits, and CitEVOL is run to identify appropriate GWAS populations (Fig. 4). From CitTRAIT, minimum (min), maximum (max), mean, standard deviation (SD), median, and coefficient of variation values (CV) can be calculated with the built-in calculator (Fig. 4a, b). With CitEVOL, SNPs of the selected species can be used for structure analysis, principal component analysis (PCA), or for the construction of phylogenetic trees with neighbor joining (NJ) model and maximum likelihood (ML) model (Fig. 4c, d). CitGWAS was designed to use trait and genetic variation data to correlate trait-related sites and genes. The results can be visualized with Manhattan plots (Fig. 4e), quantile-quantile (QQ) plots (Fig. 4f) and related SNP/gene lists as an output file. The build-in tools can be used free of charge with computation powered by the database creator. In addition to the three pipelines, tools Gbrowse and Primer Design, a BLAST tool was established to search the orthologs or paralogs of an input sequence in CitGVD. The programs blastn, blastp, blastx, tblastn and tblstx can be used with a nucleic acid or peptide as the query to retrieve hits from the genomes, CDSs and peptides of the two references.

### DOWNLOAD

All built-in data including genotypic data, phenotypic data, NGS sequencing reads and reference data can be downloaded from CitGVD free of charge by both the registered users and visitors. The register/login allows users to access and manage their own data output from the built-in pipelines in CitGVD (e.g., CitGWAS and CitEVOL). The results of “BROWSE” and “SEARCH” as well as tool CitTRAIT can be retrieved from the result pages by clicking on a responding link termed “Download” (Figs. 2b, 3b, c, 4b).

### GENERAL

In the “GENERAL” section, detailed information on the features of CitGVD, built-in data statistics, data resources, contributors, references and database usage are provided for the users. This information provides a complete understanding of the database and provides citrus researchers with an important reference for molecular breeding.

### HELP

To ensure ease of use for first-time visitors, CitGVD provides a “HELP” tab in which users can access a FAQs subpage containing answers to a range of queries and broad information regarding citrus genomics. Detailed information, including that pertaining to software tools, parameters and data sources, is provided on the Data Pipeline page. A step-by-step user-guide tutorial for CitGVD users regarding variation searches, data downloads, phenotypic analysis, evolution, GWASs, chromosomal visualization and primer design are provided. Trait evaluation pages introduce standardized approaches for trait evaluation.

## Discussion and future perspectives

Citrus evolution, traits and breeding are hot topics in biological studies. As the first database concentrating on citrus whole-genome variations (SNPs and INDELs), CitGVD stores a large volume of uniformly distributed high-quality NGS data, which compensates for the lack of citrus SNPs/INDELs provided by other researchers. With a high resequencing depth, sample coverage and accuracy from 346 individual samples, our nonredundant SNP/INDEL datasets can be used as a citrus SNP reference. Users can also perform in-depth analysis with variation and phenotypic data using built-in tools, such as CitTRAIT, CitEVOL, and CitGWAS, to assess evolutionary histories, trait-related markers and annotated genes.

CitGVD is the first genome-wide variation database for horticultural species. Compared to previous databases designed to centralize species genomic variations, such as DoGSD for dog/wolves^16^ and SorGSD for sorghum^17^, CitGVD is more powerful due to its combination of phenotypic data, genomic variations and in-depth analysis tools and pipelines which make evolutionary analysis and GWASs possible. Furthermore, CitGVD provides gene blast and annotation sections, which allow to annotate genes or gene families of *Citrus species*^30–32^. For rice, RiceVarMap^18^ includes phenotypic data and GWASs that are similar to those in CitGVD. However, the GWAS section in RiceVarMap is limited to visualizing studies performed in local computer clusters, while in CitGVD, the user can use intact GWAS pipelines with background computing resources and built-in data. The citrus genome database (CGD, https://www.citrusgenomedb.org) is a citrus-specific database housing genomics, genetics, and breeding data for citrus species and does not focus on genomic variations.

To keep up to date with whole-genome SNP data and to update CitGVD in a timely manner with information from population studies and closely related species, an uploading function can be added that allows users to directly submit WGS data or SNP lists. Once WGSs are obtained, we can identify variations through data processing pipelines and deploy CitGVD in a timely manner. In the present version, only SNPs and INDELs are included. In the updated version, we will process and integrate a greater number of genetic structural variations, including copy number variations (CNVs) and structure variations (SVs) from internal and external sources. In addition, a greater number of citrus individuals, reference genomes (e.g., *Citrus sinensis*) and phenotypes will be included. The current version of CitGVD contains genotypes (SNPs/INDELs) and phenotypes. We will further include metabolomics data and develop a new pipeline, metabolome genome-wide association studies (MGWASs), to enrich the metabolic pathways. As a long-term research project, a new tool, CitMMAS, used for citrus molecular marker assistant selection (MMAS) will be developed. With WGS and variation data for new varieties, key molecular markers and trait characteristics will be automatically predicted. A new interface for additional content, features and functions will be designed.

In summary, CitGVD serves as a bioinformatics platform to inform wet-lab experiments, including those involving biomarker development, genetic analysis and molecular breeding strategies. In addition to collaborations among the broader research community, we will collaborate with domestic and international laboratories to sequence and annotate a larger number of citrus accessions in future studies.
